# Marker-Assisted Selection at VEQ2 Increases α-Tocopherol Content in Rice Grains Without Yield Penalty

**DOI:** 10.3390/plants15192951

**Published:** 2026-09-28

**Authors:** Hongbo Yang, Yu Han, Haifeng Zhu, Ruolin Zhou, Sishu Huang, Xindong Li, Yufei Li, Jie Luo, Tuan Long

**Affiliations:** 1 School of Breeding and Multiplication (Sanya Institute of Breeding and Multiplication), School of Tropical Agriculture and Forestry, Hainan University, Sanya National Center of Technology Innovation for Saline-Alkali Tolerant Rice, Sanya 572025, China; 17724900868@163.com (H.Y.); 15234548597@163.com (Y.H.); 23220951310050@hainanu.edu.cn (H.Z.); 24220951310208@hainanu.edu.cn (R.Z.); 23220951310042@hainanu.edu.cn (X.L.); 2 Yazhouwan National Laboratory, Sanya 572025, China; huangsishu@yzwlab.cn (S.H.); liyufei@yzwlab.cn (Y.L.)

**Keywords:** rice, vitamin E, α-tocopherol, VEQ2, genome-wide association study, marker-assisted selection, biofortification, near-isogenic line

## Abstract

Vitamin E is an essential dietary nutrient, yet deficiency affects large populations, especially in rice-dependent developing countries. To dissect the genetic basis of grain vitamin E content in rice, we performed a genome-wide association study (GWAS) on a diverse panel of 533 rice accessions. A strongly associated locus, VEQ2, was identified on chromosome 2, where the CC genotype significantly increased α-tocopherol content compared with the TT genotype. We developed a kompetitive allele-specific PCR (KASP) marker for VEQ2 and validated its effect in an F_2_ population derived from Baikezaohe (TT) and AKITAKOMACHI (CC), consistent with largely additive (incomplete dominance) inheritance. Through marker-assisted backcrossing, we introgressed VEQ2 into an elite indica background, generating near-isogenic lines. The CC introgression lines exhibited α-tocopherol and α-tocotrienol levels that were approximately 1.86- and 5.28-fold those of the control, respectively, accompanied by reduced β-tocopherol, whereas the TT lines displayed the opposite metabolic profile, with no significant penalties in agronomic traits. Analysis of 4726 accessions revealed marked differences in allele frequencies between subspecies: TT was predominant in indica (84.2%), whereas CC was nearly fixed in japonica (99.5%). Collectively, these findings demonstrate that VEQ2 is a key associated locus modulating the rice vitamin E metabolic profile and represents a promising target for biofortification of indica rice without compromising yield.

## 1. Introduction

Vitamin E comprises a group of lipid-soluble antioxidants that are ubiquitously distributed in plant seeds, leaves, and animal tissues. In plants, it plays essential roles in stress tolerance, maintenance of photosynthetic membrane integrity, and seed longevity [1]. In humans, vitamin E protects cellular membranes from lipid peroxidation and modulates multiple signaling pathways—through regulation of gene and protein expression—that are involved in lipid homeostasis, inflammation, cell survival/apoptosis, angiogenesis, tumorigenesis, neurodegeneration, and senescence [2,3]. Since humans lack the ability to synthesize vitamin E de novo, it must be obtained from dietary sources. However, approximately 64% of the global population suffers from inadequate vitamin E intake, with rice-dependent developing countries being particularly affected [4]. In addition, the global market for vitamin E as an animal feed additive is valued at approximately USD 574.4 million [5]. Therefore, elucidating the biosynthetic and regulatory mechanisms of vitamin E and enhancing its content in staple crops such as rice represents one of the most cost-effective and sustainable strategies for improving human and animal nutrition.

Vitamin E (collectively termed tocochromanols) belongs to a class of amphipathic isoprenoid derivatives, each molecule consisting of a polar chromanol ring and a hydrophobic isoprenoid side chain [1]. Based on side-chain saturation, tocochromanols are classified into two major groups: tocopherols, which carry a saturated phytyl side chain, and tocotrienols, which contain an unsaturated farnesyl side chain with three trans double bonds [5,6]. Each group is further divided into α, β, γ, and δ isoforms according to the number and position of methyl groups on the chromanol ring. In addition, rare isoforms such as plastochromanol-8, which has a longer side chain, and tocomonoenols, which carry a single double bond, also exist in plants [7,8].

Among all vitamin E isoforms, α-tocopherol exhibits the highest nutritional and physiological activity, as it is preferentially absorbed, transported, and accumulated due to its highest affinity (100% relative binding efficiency) for the human hepatic α-tocopherol transfer protein [5]. By contrast, although γ-tocopherol constitutes the majority of dietary intake, its proportion in the circulatory system is far lower than that of α-tocopherol as a result of selective hepatic processing [9]. Notably, tocotrienols, owing to the unsaturated double bonds in their side chains, possess stronger membrane penetration ability and more efficient radical-scavenging activity, and they exhibit unique physiological functions, particularly in cholesterol reduction and neuroprotection [10].

The biosynthetic pathway of vitamin E in plants is a complex metabolic network governed by precursor supply, core enzymatic reactions, and multilayered regulatory mechanisms. The pathway strictly depends on two major precursors: the aromatic ring donor homogentisate (HGA) and the isoprenoid side-chain donors phytyl diphosphate (PDP) or geranylgeranyl diphosphate (GGPP). HGA is derived from the tyrosine degradation pathway, sequentially catalyzed by tyrosine aminotransferase (TAT) and 4-hydroxyphenylpyruvate dioxygenase (HPPD) [1,5]. The side-chain precursors PDP and GGPP are mainly supplied by the plastidial methylerythritol phosphate (MEP) pathway. In addition, the seed-specific alpha/beta hydrolases (ABH/VTE7), phytol kinase (PK/VTE5), and phytyl phosphate kinase (PKK/VTE6) mediate the direct recycling and reutilization of phytol from chlorophyll degradation, thus establishing a molecular bridge between chlorophyll catabolism and vitamin E biosynthesis [1,11]. Upon entry into the core synthesis stage, HGA condenses with PDP, catalyzed by homogentisate phytyltransferase (HPT/VTE2), to form 2-methyl-6-phytyl-1,4-benzoquinol (MPBQ), initiating tocopherol synthesis; whereas HGA condenses with GGPP, catalyzed by homogentisate geranylgeranyltransferase (HGGT), to form 2-methyl-6-geranylgeranylbenzoquinol (MGGBQ), initiating tocotrienol synthesis. Subsequently, MPBQ methyltransferase (MPBQ-MT/VTE3) and tocopherol cyclase (TC/VTE1) catalyze methylation and cyclization, respectively, yielding δ- and γ-tocopherols (or tocotrienols), which are ultimately converted to the most biologically active α-forms by γ-tocopherol methyltransferase (γ-TMT/VTE4) [1,5]. The pathway is also subject to multilayered regulation by transcription factors (e.g., GmZF351) and environmental stress signals (high temperature, high light, drought, etc.), enabling dynamic responses to developmental and environmental cues [5].

Despite substantial progress in elucidating the biosynthetic pathway, the genetic architecture underlying grain vitamin E content in rice remains poorly understood. *OsVTE2* is not only involved in tocopherol biosynthesis but is also essential for seed longevity, plant development, and cold tolerance [12,13]. Polymorphisms in the 3′ UTR of *OsVTE4* significantly affect α-tocopherol content in indica and japonica rice grains [14]. Two independent GWAS studies have identified 13 and 18 QTLs associated with grain vitamin E content, respectively [15,16]. *OsVTE1* (LOC_Os02g17650) has been reported to be associated with salt tolerance [17]. More recently, a transcriptome-based study identified 10 α-tocopherol biosynthesis genes in the rice genome and showed that these genes respond effectively to anilofos-induced stress, potentially promoting α-tocopherol production and modulating pesticide degradation [18]. However, with the exception of *OsVTE2* and *OsVTE4*, the contributions of other genes or loci to grain vitamin E content remain to be functionally validated and allelically characterized.

Currently, genetic improvement of vitamin E content in rice relies predominantly on transgenic approaches, whereas the application of marker-assisted selection remains underexploited. Introduction of sunflower-derived *HaHPT* and *HaTMT* into the elite rice cultivar Ningjing 7 increased grain α-tocopherol content by up to 2.33-fold in transgenic lines without significantly affecting agronomic performance, suggesting that this metabolic engineering strategy is effective for enhancing rice grain vitamin E content [19]. In the indica cultivar ASD16, single or combined introduction of *Arabidopsis AtVTE1* and *AtVTE2* increased leaf α-tocopherol content by 2.2- to 5.3-fold, whereas the increase in seed α-tocopherol content was modest, with a maximum of 1.29-fold [20].

The recommended dietary allowance (RDA) of vitamin E for adults is 15 mg α-tocopherol per day [21]. Rice is the primary caloric source for more than half of the global population; however, the α-tocopherol content of brown rice is typically below 1 mg/100 g [15], so that even a daily consumption of 300 g of rice supplies only a minor fraction of the daily requirement. Grain vitamin E is also progressively lost during post-harvest handling: milling removes most of the tocopherols concentrated in the bran and embryo [22], and further losses occur during storage and cooking [23]. These considerations indicate that even a moderate genetic increase in grain α-tocopherol, if stably expressed in the milled and cooked product, could translate into a nutritionally meaningful improvement in vitamin E intake for rice-based diets.

In this study, using a diverse panel of 533 rice accessions, we performed a genome-wide association study (GWAS) and identified VEQ2, a locus strongly associated with brown rice α-tocopherol content. We developed a kompetitive allele-specific PCR (KASP) marker for VEQ2 and validated its genetic effect in an F_2_ segregating population. Through marker-assisted backcrossing, using an elite indica cultivar as the recurrent parent and a high-α-tocopherol japonica cultivar as the donor, we generated near-isogenic introgression lines at different generations. We systematically characterized the vitamin E metabolic profiles, agronomic traits, and background genome recovery rates of these introgression lines, and we analyzed the allelic distribution patterns of VEQ2 across 4726 rice accessions. This study aims to provide elite alleles, functional molecular markers, and improved germplasm resources that are readily applicable for vitamin E biofortification in rice breeding programs.

## 2. Results

### 2.1. VEQ2 Is a Strongly Associated Locus for Grain α-Tocopherol Content in Rice

To identify genetic loci controlling grain α-tocopherol content in rice, we assembled a diverse panel of 533 rice accessions collected from worldwide sources [24]. Ultra-high-performance liquid chromatography–tandem mass spectrometry (UPLC-MS/MS) analysis revealed substantial variation in brown rice α-tocopherol content across the panel, with the highest-content accession exhibiting 91.2-fold that of the lowest-content accession, and a coefficient of variation (CV) of 61.31% (Table S1). GWAS analyses using this panel identified 12 loci associated with brown rice α-tocopherol content. Among these, the most prominent locus was located at approximately 28.8 Mb on chromosome 2, which we designated as VEQ2 (Figure 1A). The lead single nucleotide polymorphism (SNP) at the VEQ2 locus, vg0228814969 (position 28,814,969 bp on chromosome 2), was used as the representative genotype for VEQ2 in subsequent analyses. Two alleles, T and C, were identified at VEQ2. A total of 285 accessions carried the homozygous T genotype, with a mean relative α-tocopherol content of 69,528.84, whereas 247 accessions carried the homozygous C genotype, with a mean relative α-tocopherol content of 146,524.47. Student’s *t*-test revealed a highly significant difference in α-tocopherol content between the two genotypic groups (*p* = 1.38 × 10^−47^; Figure 1B). These results demonstrate that VEQ2 is a locus strongly associated with grain α-tocopherol content in rice, with the CC genotype conferring the high-content phenotype and the TT genotype conferring the low-content phenotype.

### 2.2. Development and Validation of a Marker for the VEQ2 Locus

To facilitate the utilization of VEQ2 for improving grain α-tocopherol content in rice, we first developed a KASP marker for genotyping the VEQ2 locus. Genotyping of 13 randomly selected accessions from the 533-panel using this KASP marker identified 11 TT genotypes and 2 CC genotypes (Figure 2A), which were fully consistent with Sanger sequencing results at the VEQ2 locus for the same 13 accessions (Table S2), confirming the reliability of the KASP marker for VEQ2 genotyping.

To validate the genetic effect of VEQ2, we generated an F_2_ segregating population from a cross between Baikezaohe (TT genotype) and AKITAKOMACHI (CC genotype). KASP genotyping of the F_2_ population (Table S3) identified 27 TT plants, 26 CC plants, and 63 heterozygous (CT) plants, fitting a 1:2:1 segregation ratio (χ^2^ = 0.442, *p* = 0.80), indicating that VEQ2 does not affect gamete fertility, and segregation follows Mendelian expectations.

F_2_ plants with single-plant seed yield above 10 g (for brown rice) and 20 g (for milled rice) were selected for α-tocopherol content measurement (Table S3). In brown rice, CC plants exhibited a 75.6% higher mean relative α-tocopherol content compared with TT plants (*p* = 0.019); CT plants showed a 40.5% increase over TT plants, although this difference was not statistically significant (Figure 2B). To characterize the mode of inheritance more quantitatively, we estimated the additive (a) and dominance (d) effects from the three genotypic means (a = (CC − TT)/2; d = CT − (CC + TT)/2). The resulting d/a ratio was close to zero (d/a = 0.07), indicating a predominantly additive mode of inheritance, with the heterozygote located near the parental midpoint. However, owing to the limited F_2_ population size and the large within-genotype variance, the heterozygote was not significantly different from either homozygote, and a larger segregating population will be needed to conclusively resolve the inheritance mode. In milled rice, CC and CT plants showed 69.6% and 66.1% higher mean relative α-tocopherol content than TT plants, respectively, although the differences were not significant (Figure 2C). The mean α-tocopherol content in milled rice for the three genotypes were approximately 17.7%, 18.6%, and 24.2% of those in brown rice, with no significant differences among genotypes, suggesting that the processing retention rate of α-tocopherol is not affected by genotype and that the difference in α-tocopherol content primarily originates from the initial content in brown rice (Figure 2D). Collectively, these results indicate that VEQ2 primarily affects α-tocopherol content in brown rice.

### 2.3. Development of Backcross Introgression Lines at the VEQ2 Locus Through Marker-Assisted Selection

PXB is an improved line derived from the widely cultivated indica variety Huanghuazhan in South China, carrying introgressed genes for blast resistance (*Pi1*, *Pita*, *Pi9*), bacterial blight resistance (*Xa7*, *Xa21*, *Xa23*), and brown planthopper resistance (*Bph14*, *Bph15*). AKITAKOMACHI is a high-quality japonica variety from Japan. Using PXB (TT genotype) as the recurrent parent and AKITAKOMACHI as the donor parent, we developed backcross introgression lines at the VEQ2 locus through successive crossing, backcrossing, and selfing, including 15 CC-genotype lines for the BC_2_F_2_, BC_2_F_5_, and BC_3_F_4_ generations and 5 TT-genotype lines for the BC_2_F_2_, BC_3_F_3_, and BC_3_F_4_ generations (Table S4). The plant architecture of these lines was largely similar to that of the recurrent parent PXB (Figure 3).

Background genome analysis (Table S4) revealed that the mean recurrent parent genome recovery rate for CC-genotype lines was 86.54 ± 1.87% for the BC_2_ generation (*n = 9) and increased to 93.83 ± 1.09% for the BC_3_ generation (n = 6), showing a step*wise increase. For TT-genotype lines, the recovery rate was 87.98% at BC_2_ (*n* = 1) and increased to 96.29 ± 1.17% at BC_3_ (*n* = 4), similar to the CC-genotype lines for the corresponding generations. Within each generation, the CV among lines was low (range 1.16–2.16%), indicating consistent background recovery among lines of the same generation.

### 2.4. CC-Genotype Introgression Lines Exhibit Significant Enrichment of α-Type Vitamin E Components

Using PXB (TT genotype) as the control, we quantified vitamin E components in brown rice of 15 CC-genotype introgression lines (Table S4). As shown in Figure 4A, α-tocopherol content in CC-introgression lines was significantly elevated compared with PXB (42.47 μg/g), with a mean of 1.86-fold that of PXB (range 1.42–2.35-fold). Among these, three BC_3_F_4_ lines—LT37 (99.88 μg/g), LT36 (95.35 μg/g), and LT34 (93.80 μg/g)—reached or exceeded the level of the donor parent AKITAKOMACHI (92.41 μg/g; 2.18-fold).

The increase in α-tocotrienol was even more pronounced, with CC-introgression lines exhibiting a mean of 5.28-fold that of PXB (range 4.00–6.43-fold; Figure 4B). Two lines, LT57 (6.43-fold) and LT60 (6.33-fold), showed high α-tocotrienol accumulation comparable to AKITAKOMACHI (6.17-fold).

Concurrently, β-tocopherol content in CC-introgression lines was significantly reduced compared with PXB, with a mean of only 0.66-fold (range 0.46–0.92-fold; Figure 4C), even lower than the 0.81-fold level of AKITAKOMACHI, suggesting that backcross introgression may further suppress β-tocopherol accumulation in the CC background. β-Tocotrienol content remained at approximately 0.85-fold that of PXB (range 0.70–1.04-fold; Figure 4D), showing a slight overall decreasing trend. δ-Tocotrienol content showed a mean of 1.33-fold that of PXB (range 0.85–1.83-fold; Figure 4E), with LT57 exhibiting the highest level at 1.83-fold.

### 2.5. TT-Genotype Introgression Lines Display a “High-β, Low-α” Vitamin E Profile

In contrast to the CC-genotype introgression lines, the five TT-genotype introgression lines exhibited a distinctly different vitamin E profile (Figure 4; Table S4). In TT lines, α-tocopherol content showed no significant difference from PXB, averaging 1.09-fold (range 1.03–1.15-fold; Figure 4A); α-tocotrienol content was also comparable to PXB, with a mean of 1.17-fold (range 0.96–1.27-fold; Figure 4B). Notably, β-tocopherol content was significantly elevated compared with PXB, averaging 1.30-fold (range 1.19–1.43-fold; Figure 4C), with LT42 showing the highest value (1.43-fold). β-Tocotrienol content also increased significantly, with a mean of 1.22-fold (range 1.12–1.31-fold; Figure 4D). δ-Tocotrienol content averaged 1.20-fold but displayed considerable variation among lines (range 0.86–1.75-fold; Figure 4E), with LT58 showing the highest level (1.75-fold), whereas LT48 (0.86-fold) and LT49 (0.87-fold) fell below the PXB level. These results reveal that, under a similar genetic background, the CC and TT genotypes generate opposite vitamin E accumulation profiles.

### 2.6. Intergenerational Stability of Vitamin E Profiles in Backcross Introgression Lines

To evaluate the genetic stability of the target traits across different backcross generations, we compared the introgression lines grouped by generation: BC_2_F_2_, BC_2_F_5_, BC_3_F_3_, and BC_3_F_4_ (Table S5). For CC-genotype lines, the mean α-tocopherol content for BC_2_F_2_, BC_2_F_5_, and BC_3_F_4_ were 86.75 μg/g, 74.77 μg/g, and 72.70 μg/g, respectively, with an intergenerational CV of only 9.71%, indicating stable expression. The intergenerational CVs for α-tocotrienol, β-tocotrienol, and δ-tocotrienol were 7.70%, 6.25%, and 3.28%, respectively, all within highly stable ranges. β-Tocopherol showed relatively higher intergenerational variation (CV = 14.79%), though still within an acceptable range.

For TT-genotype lines, α-tocopherol (CV = 3.71%), α-tocotrienol (CV = 8.56%), β-tocopherol (CV = 0.69%), and β-tocotrienol (CV = 6.33%) all exhibited high stability across the BC_2_F_2_, BC_3_F_3_, and BC_3_F_4_ generations. δ-Tocotrienol showed greater intergenerational variation (CV = 34.6%), primarily due to the lower mean value in the BC_3_F_3_ generation. Overall, with the exception of δ-tocotrienol in the TT background, all major vitamin E components exhibited good genetic stability across the BC_2_ to BC_3_ generations, indicating that the high vitamin E traits conferred by the target genotypes have been effectively fixed during backcross introgression.

### 2.7. The VEQ2 Locus Does Not Affect Other Agronomic Traits

To evaluate the potential pleiotropic effects of the VEQ2 locus on other agronomic traits, we systematically measured plant height, tiller number, seed setting rate, grain shape, and yield-related traits in the 20 backcross introgression lines (Table 1). Although individual lines showed varying degrees of difference from PXB for certain traits, the mean values of both CC- and TT-genotype lines were generally similar to those of PXB (Table 1). CC-genotype lines (*n* = 15) had a mean plant height of 92.91 ± 5.23 cm (range 86.86–103.29 cm), comparable to PXB (100.7%). TT-genotype lines (*n* = 5) had a mean plant height of 95.43 ± 3.85 cm (range 89.71–98.00 cm), slightly higher than PXB (103.4%). CC-genotype lines showed a mean tiller number of 14.77 ± 1.77 (range 12.43–17.71), representing an 18.8% increase over PXB (12.43) and a 16.2% increase over AKITAKOMACHI (12.71). TT-genotype lines had a mean tiller number of 13.75 ± 0.72, a 10.6% increase over PXB. For seed setting rate, CC-genotype lines averaged 79.92 ± 2.57% (range 75.93–87.65%), approximately 2.41 percentage points higher than PXB (77.51%). TT-genotype lines averaged 79.12 ± 1.45%, comparable to the CC lines.

Grain shape varied significantly across generations among CC-genotype lines. BC_3_F_4_ lines exhibited grain length (7.78 ± 0.40 mm), grain width (1.86 ± 0.10 mm), and length-to-width ratio (4.33 ± 0.05) close to or exceeding those of PXB. However, BC_2_F_2_ and BC_2_F_5_ lines showed significantly reduced grain length (6.50 mm and 4.56 mm, respectively), narrower grain width (1.83 mm and 1.53 mm), and markedly reduced length-to-width ratios (3.60 and 3.02). TT-genotype lines displayed stable grain shape traits highly consistent with PXB. The mean 1000-grain weight of CC- and TT-genotype lines was 21.11 ± 0.10 g and 21.15 ± 0.08 g, respectively, essentially identical to PXB (21.16 g). CC-genotype lines had a mean yield per plant of 25.85 ± 2.09 g (range 22.72–28.75 g), representing 98.7% of PXB (26.19 g), with LT37 showing the highest yield (28.75 g), significantly exceeding PXB. TT-genotype lines had a mean yield per plant of 25.12 ± 3.43 g, representing 95.9% of PXB, with LT58 showing the highest yield (27.52 g).

### 2.8. Genotype Distribution of the VEQ2 Locus in Rice Germplasm Resources

To characterize the natural variation pattern of the VEQ2 locus across different rice subspecies, we analyzed the genotype distribution of the VEQ2 locus in 4726 rice accessions (Table 2). The VEQ2 locus exhibited two major genotypes, TT and CC, accounting for 52.7% and 46.9% of the total population, respectively. At the subspecies level, marked differences in allele frequencies between the two subspecies were observed. Among 2759 indica accessions, the TT allele was overwhelmingly predominant, with a frequency of 84.2%, whereas the CC allele accounted for only 15.3%. In contrast, among 1512 japonica accessions, the CC allele was nearly fixed, with a frequency of 99.5%, while the TT allele was extremely rare (0.4%). In the aus group (*n* = 269), the TT and CC alleles were present at near-equilibrium frequencies of 49.8% and 50.2%, respectively. The VI/Aromatic group (*n* = 96) resembled japonica in having a predominance of the CC allele (99%), with TT accounting for only 1%. Among admixture accessions (*n* = 90), the CC allele frequency was 67.8%, while TT accounted for 28.9%.

To evaluate whether the allelic differentiation at VEQ2 reflects locus-specific selection or the genome-wide population structure between the two subspecies, we calculated the Weir–Cockerham weighted fixation index (*F_ST_*) and nucleotide diversity (π) in 10 kb sliding windows (1 kb step) around VEQ2 and at three comparative points—SH4 and BADH2 (two well-characterized domestication genes controlling seed shattering [25] and grain fragrance [26], respectively) and a genomic background position (chr01:10,000,000)—using genotype data of the 4726 accessions (RiceVarMap v3.0) (Table S6; Figure 5).

The *F_ST_* between indica and japonica at VEQ2 (0.653) exceeded that at *SH4* (0.290) and *BADH2* (0.439) but fell below the genome-wide 95th percentile (0.8736); the background position showed a comparable *F_ST_* (0.640), indicating that the strong, genome-wide differentiation between the subspecies precludes *F_ST_*-based inference of selection at VEQ2 (Table S6). In contrast, nucleotide diversity revealed a distinctive pattern: at VEQ2, the π_indica/π_japonica ratio was 4.28 (π_indica = 0.00277; π_japonica = 0.00065), approximately twice the genome-wide background benchmark (~2.16, derived from the 95th percentiles of π in indica and japonica), and far above the *BADH2* ratio (2.06) and *SH4* ratio (0.44, reflecting reduced diversity in the indica lineage). Together with the near-fixation of the CC genotype in japonica (99.5%), these data are consistent with a japonica-lineage-specific selective sweep at the VEQ2 locus.

## 3. Discussion

### 3.1. VEQ2 Is a Novel Locus Strongly Associated with Grain α-Tocopherol Content in Rice

A recent genome-wide association study has identified 13 QTLs associated with grain α-tocopherol and total tocopherol content in rice, distributed across chromosomes 1, 2, 3, 6, and 8 [15]. In the present study, using a diverse panel of 533 rice accessions, we identified a novel locus, VEQ2, on chromosome 2 that is strongly associated with brown rice α-tocopherol content (Figure 1A). Genotyping based on the lead SNP vg0228814969 revealed that accessions carrying the CC genotype exhibited significantly higher α-tocopherol content than those with the TT genotype (*p* = 1.38 × 10^−47^), with an approximately 110% increase (Figure 1B; Table S1). These results establish VEQ2 as a previously unreported locus strongly associated with grain α-tocopherol content in rice.

Among the previously reported QTLs, five loci mapped to chromosome 2 in two recent GWAS of grain tocopherol content [15,16]—qTol2.1 for total tocopherol, qAlph2.1 for α-tocopherol, qDelt2.1 for δ-tocopherol, qDelt2.2 for δ-tocopherol, and qGam2.1 for γ-tocopherol—are located at approximately 4.66 Mb, 9.49 Mb, 35.11 Mb, 28.90 Mb, and 35.12 Mb, respectively. qTol2.1 and qAlph2.1 explained only 9% and 7% of the phenotypic variation, respectively, and the other three showed weak association signals (−log_10_(*p*) = 4.6–6.9). VEQ2 (chr02:28,814,969) is separated from qTol2.1, qAlph2.1, qDelt2.1, and qGam2.1 by 6.3–24.2 Mb of physical distance and does not overlap these loci. The remaining chromosome 2 locus, qDelt2.2, lies only approximately 87 kb from VEQ2 and thus falls within the same candidate region; however, qDelt2.2 was detected for δ-tocopherol with weak statistical support (−log_10_(*p*) = 4.6) in a panel of 179 accessions genotyped with 34,323 SNPs. Moreover, VEQ2 showed a large and highly significant effect (an approximately 110% increase of CC over TT; *p* = 1.38 × 10^−47^) that was consistently validated in an F_2_ population and in backcross-derived near-isogenic lines. Whether qDelt2.2 and VEQ2 represent the same causal locus or distinct linked loci within this candidate region will require fine-mapping to resolve.

The genetic effect of VEQ2 was further validated in an F_2_ population derived from Baikezaohe (TT) × AKITAKOMACHI (CC). In brown rice, CC homozygotes displayed 75.6% higher α-tocopherol content than TT homozygotes, with heterozygotes showing intermediate levels (Figure 2B; Table S3), consistent with a largely additive (incomplete dominance) inheritance pattern. This dosage-dependent effect suggests that even a single copy of the C allele can partially enhance α-tocopherol accumulation. Notably, while CC and CT plants both exhibited higher α-tocopherol content in milled rice than TT plants, the differences were not statistically significant, indicating that the VEQ2 locus functions primarily in brown rice—likely in the embryo, pericarp and/or aleurone layers—with limited contribution to the endosperm.

It should be emphasized that vg0228814969 is the lead SNP identified by GWAS, and its status as the causal variant has not been formally established. The true causal variant may reside in linkage disequilibrium with vg0228814969 and could involve regulatory or coding changes in nearby genes. Fine-mapping through high-resolution association mapping, haplotype analysis, and functional validation (e.g., gene editing or transgenic complementation) will be required to establish causality.

### 3.2. Allelic Variation at VEQ2 Remodels the Vitamin E Metabolic Profile

Previous studies have shown that changes in seed α-tocopherol content in plants are often accompanied by reciprocal changes in γ-tocopherol [14,27]. The most striking finding of the present study is that, under a near-isogenic background, the CC and TT genotypes at VEQ2 produced opposite vitamin E metabolic profiles. In CC-introgression lines, we observed significant enrichment of α-tocopherol (1.86-fold) and α-tocotrienol (5.28-fold), accompanied by a marked reduction in β-tocopherol (0.66-fold) compared with the recurrent parent PXB (Figure 4; Table S4). In contrast, TT-introgression lines showed no significant differences in α-tocopherol and α-tocotrienol relative to PXB, but they exhibited significantly elevated β-tocopherol (1.30-fold) and β-tocotrienol (1.22-fold). This see-saw metabolic pattern strongly suggests that VEQ2 modulates metabolic flux through the vitamin E biosynthetic pathway, likely by influencing substrate partitioning or enzyme activity at key branch points. Future fine-mapping and functional characterization of the causal gene at VEQ2—including transcriptomic profiling and enzyme activity assays—will be essential to elucidate the precise molecular mechanism underlying this metabolic switch.

### 3.3. VEQ2 Enhances Nutritional Quality Without Compromising Yield Potential

A key concern in biofortification breeding is the potential for linkage drag associated with the introgression of donor alleles. Our results demonstrate that the C allele from the japonica donor AKITAKOMACHI was successfully introgressed into the elite indica recurrent parent PXB via marker-assisted backcrossing, with minimal impact on agronomic performance. Evaluation of 20 introgression lines revealed that plant height, tiller number, seed setting rate, grain shape, 1000-grain weight, and yield per plant were generally comparable to those of PXB (Table 1). Although individual lines showed significant deviations for certain traits—for example, reduced grain length in BC_2_F_2_ and BC_2_F_5_ CC lines, which reverted to normal in BC_3_F_4_—these variations appear to reflect transient linkage drag that can be effectively broken with additional backcrossing. The mean yield per plant of CC and TT introgression lines reached 98.7% and 95.9% of PXB, respectively, with some lines (e.g., LT37, 28.75 g; LT58, 27.52 g) even outperforming the recurrent parent. These findings indicate that VEQ2-mediated vitamin E enhancement can be achieved without yield penalty—a prerequisite for the commercial deployment of biofortified varieties.

The nutritional significance of the VEQ2-mediated increase in α-tocopherol can be appreciated by comparison with the recommended dietary allowance (RDA) of vitamin E for adults, which is 15 mg α-tocopherol per day [21]. The recurrent parent PXB contains 42.47 μg/g α-tocopherol in brown rice, whereas the CC near-isogenic lines accumulated, on average, 1.86-fold that of PXB (range 1.42–2.35-fold), corresponding to approximately 60–100 μg/g. Assuming a daily rice consumption of 300 g (brown-rice basis) for adults in rice-dependent regions, the VEQ2 CC lines would provide approximately 18–30 mg α-tocopherol per day before processing, well above the RDA and up to more than twice the contribution of the PXB control (12.7 mg/day).

Grain vitamin E is subject to substantial losses along the food chain. Milling is the most critical step, because tocopherols are concentrated in the bran and embryo: reported losses of α-tocopherol reach ~75% when brown rice is milled to white rice [28], with losses of total tocopherols of 84–90% reported at full polishing [22]. Further losses of 14.5–90% of total tocols can occur during subsequent storage and cooking [23,29], and storage of brown rice at room temperature alone reduced total tocopherols by ~70% within six months [23]. Because the lipid-rich bran and embryo—the compartments in which vitamin E is concentrated—are largely removed during milling, storage-dependent vitamin E degradation is expected to proceed more slowly in milled rice than in brown rice, although quantitative data for stored milled rice remain scarce. Even under conservative assumptions (75% milling loss followed by 50% cooking retention), the α-tocopherol delivered by 300 g of cooked milled product from the CC lines would be approximately 2.3–3.8 mg/day, compared with approximately 1.6 mg/day from the PXB control—a roughly twofold increase in the most relevant dietary form of vitamin E. Because populations at risk of vitamin E deficiency consume predominantly milled rice and often exceed 300 g/day, this increment represents a biologically meaningful contribution toward the RDA. The concomitant 5.28-fold increase in α-tocotrienol further raises total vitamin E intake. Taken together, the VEQ2 allele provides a realistic route to vitamin E biofortification that remains nutritionally significant after realistic processing losses.

### 3.4. Subspecies Differentiation at VEQ2 and Implications for Germplasm Utilization

Analysis of 4726 rice accessions revealed significant allelic differentiation at the VEQ2 locus: the TT genotype predominated in indica (84.2%), whereas the CC genotype was nearly fixed in japonica (99.5%) (Table 2). The aus subpopulation exhibited near-equilibrium frequencies of TT and CC (49.8% vs. 50.2%), consistent with previous reports that aus has an independent domestication origin and a distinct genetic basis [30,31].

From a breeding perspective, the near-fixation of the CC allele in japonica implies that japonica germplasm already harbors the high-α-tocopherol haplotype, and further improvement could focus on pyramiding VEQ2 with other minor QTLs. Conversely, the overwhelming predominance of the TT allele in indica highlights a substantial biofortification opportunity: introduction of the CC allele into elite indica varieties could significantly enhance α-tocopherol content in the most widely consumed rice subspecies globally. The KASP marker developed in this study can be readily deployed in breeding pipelines for foreground selection, enabling rapid conversion of TT to CC genotypes without the need for extensive phenotypic screening.

We further assessed whether the observed allelic differentiation at VEQ2 reflects adaptive selection related to grain vitamin E content or is primarily attributable to demographic divergence between indica and japonica. *F_ST_* alone is uninformative at this locus: its value (0.653) lies well below the genome-wide 95th percentile (0.8736) and is matched by a random genomic position (0.640), consistent with the strong, genome-wide differentiation between the two subspecies in cultivated rice [32]. By this measure, the VEQ2 allele frequency differences could plausibly arise from demographic history alone.

Nucleotide diversity, however, provides discriminating evidence. The π_indica/π_japonica ratio at VEQ2 (4.28) is approximately twice the genome-wide background benchmark (~2.16), placing VEQ2 well outside the range observed at the background position and the two domestication genes. In contrast, *SH4* shows a reversed ratio (0.44), consistent with positive selection on the indica lineage, while *BADH2* (2.06) tracks the background. We therefore infer that the japonica lineage experienced a locus-specific reduction of diversity at VEQ2 beyond what genome-wide demographic structure predicts—consistent with selection, whether direct (for vitamin E content or linked traits) or through japonica breeding bottlenecks. Definitive resolution will require sweep statistics (e.g., Tajima’s D), haplotype-based tests (e.g., integrated haplotype score), and functional validation.

### 3.5. Stability of Vitamin E Traits Across Backcross Generations

Previous studies have reported high heritability for α-tocopherol content [15]. In the present study, intergenerational stability analysis (Table S5) demonstrated that the vitamin E profiles of both CC and TT introgression lines remained highly consistent from BC_2_ to BC_3_ (with intergenerational CVs below 10% for most components), indicating that the VEQ2 effect is genetically stable and minimally influenced by background genetic noise. Notably, this stability contrasts with the transient grain-shape variations observed in BC_2_F_2_ and BC_2_F_5_ CC lines, further supporting the conclusion that VEQ2 exerts a specific and stable effect on vitamin E metabolism. The larger intergenerational variation observed for δ-tocotrienol in TT lines (CV = 34.6%) may reflect the lower mean value in the BC_3_F_3_ generation, compounded by the small sample size (*n* = 5) and the inherent sensitivity of δ-tocotrienol to environmental or epistatic effects. Nevertheless, the core traits—α-tocopherol and α-tocotrienol in CC lines, and β-tocopherol in TT lines—showed remarkable stability, suggesting that breeders can confidently select for these traits at the BC_3_ generation without concern for inter-generational instability.

### 3.6. Limitations and Future Perspectives

Several limitations of this study should be acknowledged. First, although we have established VEQ2 as a large-effect locus and validated its allelic effects, the causal gene underlying the locus has not yet been cloned. Fine-mapping to identify the causal polymorphism, followed by transgenic complementation or CRISPR-Cas9-based gene editing, will be essential to confirm the molecular function of VEQ2. Second, the TT-introgression lines comprised only five lines across three generations, limiting the statistical power for evaluating the stability of TT-associated vitamin E profiles. Third, the vitamin E analyses of the introgression lines were conducted under a single environmental condition; multi-location field trials are needed to assess the environmental stability of the VEQ2 effect. Likewise, the GWAS in this study was performed once, using grain metabolic data collected from a single growing season at a single location (Wuhan); whether the α-tocopherol variation observed among the 533 accessions is consistent and stable across different locations and years therefore remains to be validated in multi-environment trials.

Looking forward, pyramiding VEQ2 with other QTLs that control vitamin E components—such as those modulating γ-tocopherol or tocotrienol ratios—could enable rational design of rice vitamin E profiles tailored to specific nutritional or industrial applications. Furthermore, the KASP marker developed in this study is immediately applicable in breeding programs to accelerate the development of high-α-tocopherol rice varieties, contributing to global efforts to combat vitamin E deficiency through staple crop biofortification.

## 4. Materials and Methods

### 4.1. Plant Materials and Growth Conditions

A total of 533 rice accessions collected from worldwide sources were used in this study [24]. An F_2_ population derived from a cross between Baikezaohe and AKITAKOMACHI was generated for functional validation of the VEQ2 locus. Using PXB as the recurrent parent and AKITAKOMACHI as the donor parent, backcross introgression lines at the VEQ2 locus were developed through successive crossing, backcrossing, and selfing.

The 533-accession panel used for GWAS was grown in Wuhan in a single growing season, and their grain samples were used for α-tocopherol profiling as the phenotypic input for the association analysis. All the other rice materials used in this study were planted in Hainan. From 2020 to 2022, these materials were planted at the Luoniushan Experimental Station of Hainan University in Haikou during the main cropping season and at the Lingshui or Sanya bases of Guangling Hi-Tech Co., Ltd. (Lingshui and Sanya, China) during the off-season nursery. From 2023 to 2025, they were planted at the Batou Experimental Base of Sanya Nanfan Research Institute, Hainan University during the early season, and at the Dongfang Base of Hainan Nongle Nanfan Technology Co., Ltd. (Dongfang, China) during the late season. The planting spacing was 20 cm between rows and 20 cm between plants. Standard water and fertilizer management practices were applied.

### 4.2. Metabolic Sample Preparation and Profiling

Metabolic sample preparation was performed as follows: dried brown rice and milled rice were ground into powder using a mixer mill (MM400, Retsch, Haan, Germany) at 30 Hz for 60 s. Approximately 0.05–0.08 g of powder was weighed and mixed on ice in the dark with extraction solvent (methanol:acetonitrile:water = 2:2:1, *v*/*v*/*v*) at 10,000 μL per gram of sample (i.e., sample mass in g × 10,000 μL). The mixture was vortexed for approximately 15 s and then subjected to ultrasonication for 10 min, repeated three times. After centrifugation at 12,000 rpm at 4 °C for 10 min, the supernatant was filtered through a 0.22 μm pore size membrane filter (SCAA-104, ANPEL, Shanghai, China). A 2 μL aliquot of the filtered supernatant was analyzed using an UPLC-MS/MS-based targeted method as previously described by Yang et al. [33].

### 4.3. Absolute Quantification of α-Tocopherol

Aliquots of 0.2, 0.4, 0.6, and 0.8 μL of 10 mmol/L α-tocopherol standard (Cat. No. T90262, Shanghai Yuanye Bio-Technology Co., Ltd., Shanghai, China) were dissolved in 1000 μL of DMSO. A 2 μL aliquot of each diluted standard was subjected to UPLC-MS/MS analysis. A standard curve was constructed with the chromatographic peak area as the *Y*-axis and the absolute concentration of the standard as the *X*-axis, yielding the equation y = 68,610x − 20,948 (R^2^ = 0.9924). The absolute α-tocopherol content in brown rice and milled rice was calculated based on the standard curve and the dilution factor of the samples.

### 4.4. Genome-Wide Association Study

The GWAS analyses were performed using the efficient mixed-model association expedited (EMMAX) method [34]. In total, 4,300,150 high-quality SNPs with minor allele frequency (MAF) > 0.05 and deletion rate < 0.1 were selected from the 533 rice accessions for GWAS. The kinship matrix was constructed using the emmax-kin program.

### 4.5. KASP Marker Development, Detection, and Validation

Based on the KASP principle [35], we designed KASP primers for the lead SNP vg0228814969 at the VEQ2 locus. The primer sequences are listed in Table S7. KASP genotyping was performed using the KASP-TF V4.0 2× Master Mix kit (Cat. No. KBS-1050-102, LGC Biosearch Technologies, Hoddesdon, Hertfordshire, UK) on an ABI QuantStudio 7 Flex Real-Time PCR system (Applied Biosystems, Foster City, CA, USA), following the manufacturer’s instructions. Each KASP reaction was carried out in a total volume of 5 μL, containing 2.5 μL of KASP 2× Master Mix, 1 μL of genomic DNA template (50 ng/μL), 0.07 μL of primer mix (allele-specific forward primers Q2-F1 and Q2-F2 at 36 μM each, and common reverse primer Q2-R at 90 μM), and 1.43 μL of sterile double-distilled water. The thermal cycling program was: 94 °C for 15 min; followed by 10 touchdown cycles of 94 °C for 20 s and 61–55 °C for 60 s (decreasing by 0.6 °C per cycle); followed by 26 cycles of 94 °C for 20 s and 55 °C for 60 s, and the cycle number at this stage may be adjusted as required.

To further validate the KASP genotyping results, primers flanking the vg0228814969 site were designed for PCR amplification and Sanger sequencing. The primer sequences are listed in Table S7. The PCR reaction mixture (25 μL) contained 12.5 μL of 2× Taq PCR Master Mix (Cat. No. PC1120, Beijing Solarbio Science & Technology Co., Ltd., Beijing, China), 2 μL of DNA template (50 ng/μL), 1.25 μL each of primers VEQ2F and VEQ2R (10 μmol/L), and 8 μL of ddH_2_O. The PCR program was as follows: 94 °C for 5 min; 30 cycles of 94 °C for 30 s, 58 °C for 30 s, and 72 °C for 40 s; followed by 72 °C for 5 min and 25 °C for 1 min. The amplified products were sequenced by Beijing Tsingke Biotech Co., Ltd. Hainan Branch (Sanya, China).

### 4.6. Background Genome Analysis

For each backcross introgression line, five individual plants were randomly selected, and leaf samples were submitted to Huazhi Biotech Co., Ltd. (Changsha, China) for background genome analysis using the rice 1K mGPS liquid-phase chip. The analysis procedure was briefly as follows: Genomic DNA was extracted using the magnetic bead method, and sequencing libraries were constructed. Qualified libraries were subjected to paired-end 150 bp high-throughput sequencing on the DNBSEQ-T7 platform (MGI Tech Co., Ltd., Shenzhen, China). Raw sequencing data were quality-assessed using FastQC (v0.12.1), and reads containing adapter sequences or with base quality below Q20 were removed using Trimmomatic (v0.39). High-quality clean reads were aligned to the rice reference genome using the MEM algorithm of Burrows-Wheeler Aligner (v0.7.17). Variant calling was performed using FreeBayes software (v1.3.x) with parameters set as MAF ≥ 0.05 and sequencing depth ≥ 10×. Finally, the detected variants were manually verified using the Integrative Genomics Viewer (2.16.0) to ensure the accuracy of variant detection.

### 4.7. Agronomic Trait Evaluation

For each evaluated line, more than 60 plants were planted. Five sampling points were evenly selected within each plot, and 2–3 healthy plants were chosen from each sampling point for measurement of plant height, tiller number, seed setting rate, grain length, grain width, length-to-width ratio, 1000-grain weight, and yield per plant.

### 4.8. Distribution Frequency Analysis of Genotypes at the VEQ2 Locus

The allele distribution frequencies of SNP vg0228814969 were analyzed online across 4726 rice accessions from different population groups using the “Search for Variation Information with Variation ID” function on the RiceVarMap v3.0 website (https://ricevarmap.ncpgr.cn/v3/ (accessed on 18 June 2026)) [36,37].

Population genetic statistics were calculated from publicly available SNP data of 4726 rice accessions (RiceVarMap v3.0). *F_ST_* (Weir–Cockerham weighted) and nucleotide diversity (π) were computed in 10 kb sliding windows (1 kb step). Locus-level estimates represent the mean across all windows overlapping the target: the VEQ2 lead SNP vg0228814969 (chr02:28,814,969), the *SH4* gene body (chr04:34,231,186–34,233,373), the *BADH2* gene body (chr08:20,379,794–20,386,061), and a genomic background position (chr01:10,000,000). Genome-wide benchmarks were derived from the 95th percentiles of the corresponding genome-wide distributions (*F_ST_*: 0.8736; π in indica: 3.500 × 10^−4^; π in japonica: 1.621 × 10^−4^), yielding an approximate background π_indica/π_japonica ratio of 2.16.

## 5. Conclusions

In this study, we identified VEQ2, a novel locus strongly associated with grain α-tocopherol content in rice through genome-wide association analysis. The CC allele at this locus significantly increased brown rice α-tocopherol content by approximately 2.1-fold compared with the TT allele and showed largely additive (incomplete dominance) inheritance. Using a newly developed KASP marker for marker-assisted backcrossing, we successfully introgressed VEQ2 into an elite indica background and generated near-isogenic lines. The CC-introgression lines displayed a high-α, low-β vitamin E profile, with α-tocopherol and α-tocotrienol content increased by 86% and 428%, respectively, whereas the TT-introgression lines showed the opposite pattern. Agronomic evaluation revealed that the introgressed lines were comparable to the recurrent parent in yield and major traits, with no significant linkage drag detected. Germplasm analysis revealed marked differences in VEQ2 allele frequencies between indica and japonica, with the TT allele being overwhelmingly predominant in indica.

Collectively, these findings establish VEQ2 as a functionally relevant locus modulating the rice vitamin E metabolic profile and a promising target for nutritional improvement of indica rice grains. Marker-assisted selection for the favorable allele of VEQ2 enables the directional enhancement of brown rice α-tocopherol content while maintaining desirable agronomic performance, providing a valuable genetic resource and a feasible technical pathway for the molecular design breeding of functional rice.

## 6. Patents

The KASP marker at the VEQ2 locus has been granted a Chinese invention patent (patent number: ZL202211647049.7).

## Figures and Tables

**Figure 1:**
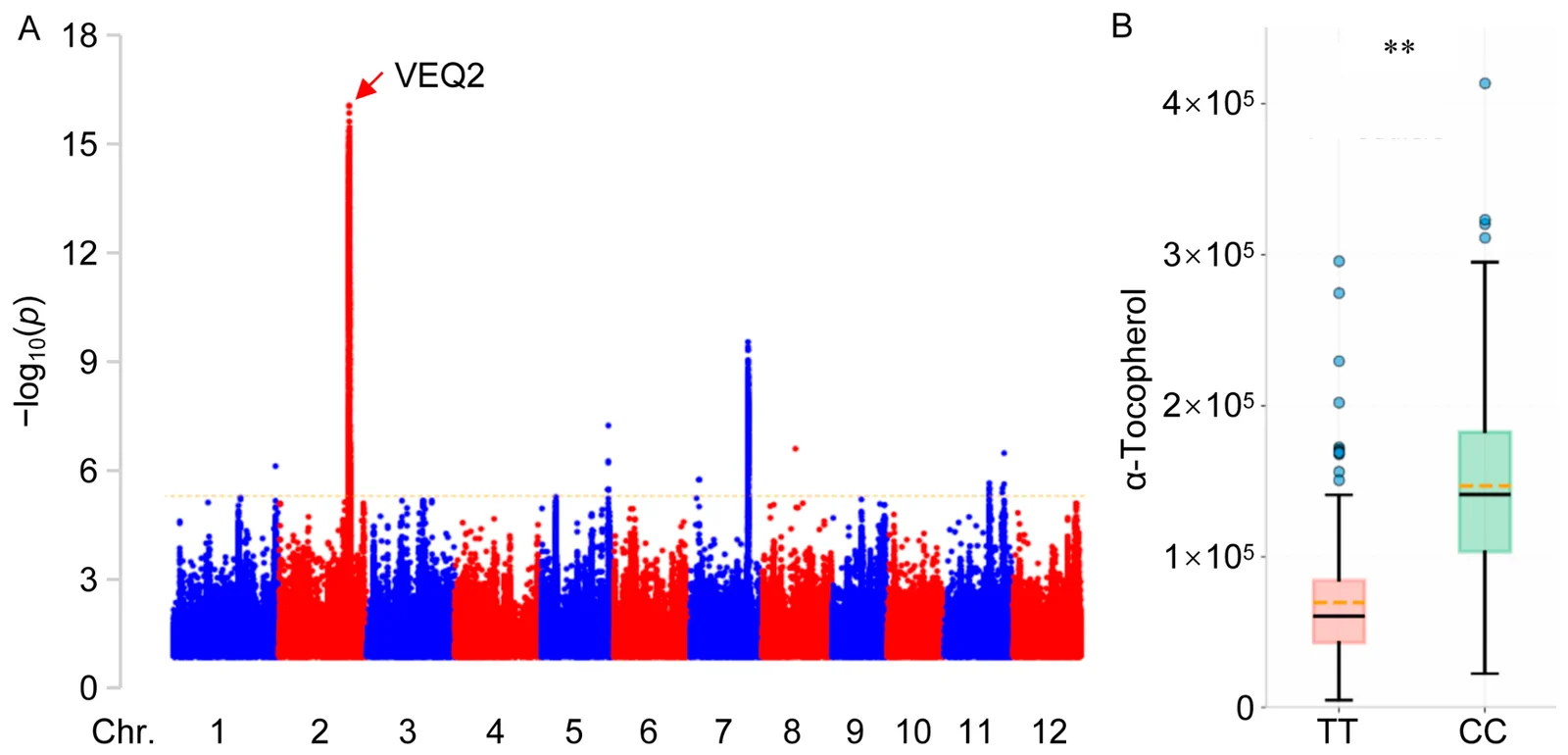
Association analysis of the VEQ2 locus with brown rice α-tocopherol content. (**A**) Manhattan plot of genome-wide association study (GWAS) for α-tocopherol content in brown rice across 533 rice accessions. The yellow dashed line indicates the significance threshold (−log_10_(*p*) = 5). Red and blue colors alternate by chromosome for visual clarity and have no statistical meaning. The arrow points to the significant association signal of the VEQ2 locus on chromosome 2. (**B**) Box plot of brown rice α-tocopherol content for VEQ2 genotypes TT (homozygous T; *n* = 285), and CC (homozygous C; *n* = 247). The upper and lower boundaries of the box represent the third quartile and first quartile, respectively. The black solid line indicates the median, the yellow dashed line indicates the mean, whiskers extend to the most extreme values within 1.5× the interquartile range, and blue dots represent outliers. **, *p* < 0.01.

**Figure 2:**
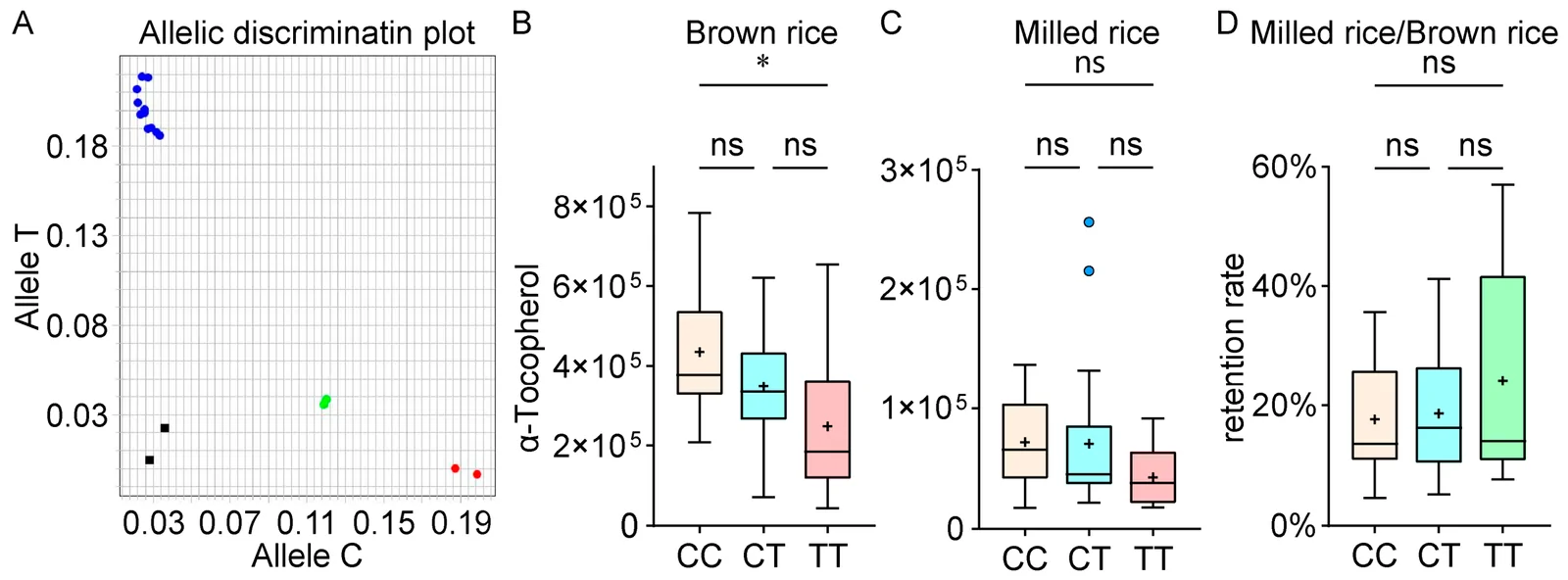
VEQ2 allelic discrimination and association of VEQ2 genotypes with α-tocopherol content in brown rice, milled rice, and retention rate in the F_2_ population of Baikezaohe × AKITAKOMACHI. (**A**) Allelic discrimination plot of the VEQ2 locus. Blue, green, and red dots represent homozygous T (TT), heterozygous (CT), and homozygous C (CC) genotypes, respectively. Black dots indicate signals of ddH_2_O. (**B**–**D**) Box plots showing α-tocopherol content in brown rice (**B**), milled rice (**C**), and the retention rate of α-tocopherol in milled rice relative to brown rice (**D**) across three VEQ2 genotypes (CC, CT, and TT) in the F_2_ population. The horizontal line within each box represents the median, the box edges indicate the 25th and 75th percentiles, and the whiskers extend to 1.5× the interquartile range. Outliers are shown as colored dots. The plus sign (+) denotes the mean. Statistical significance between genotypes was determined by one-way ANOVA followed by Tukey’s multiple comparison test. *, *p* < 0.05; ns, no significant difference. The number of samples analyzed was *n* = 13 for CC, *n* = 28 for CT, and *n* = 9 for TT in brown rice; *n* = 11 for CC, *n* = 24 for CT, and *n* = 9 for TT in milled rice and retention rate.

**Figure 3:**
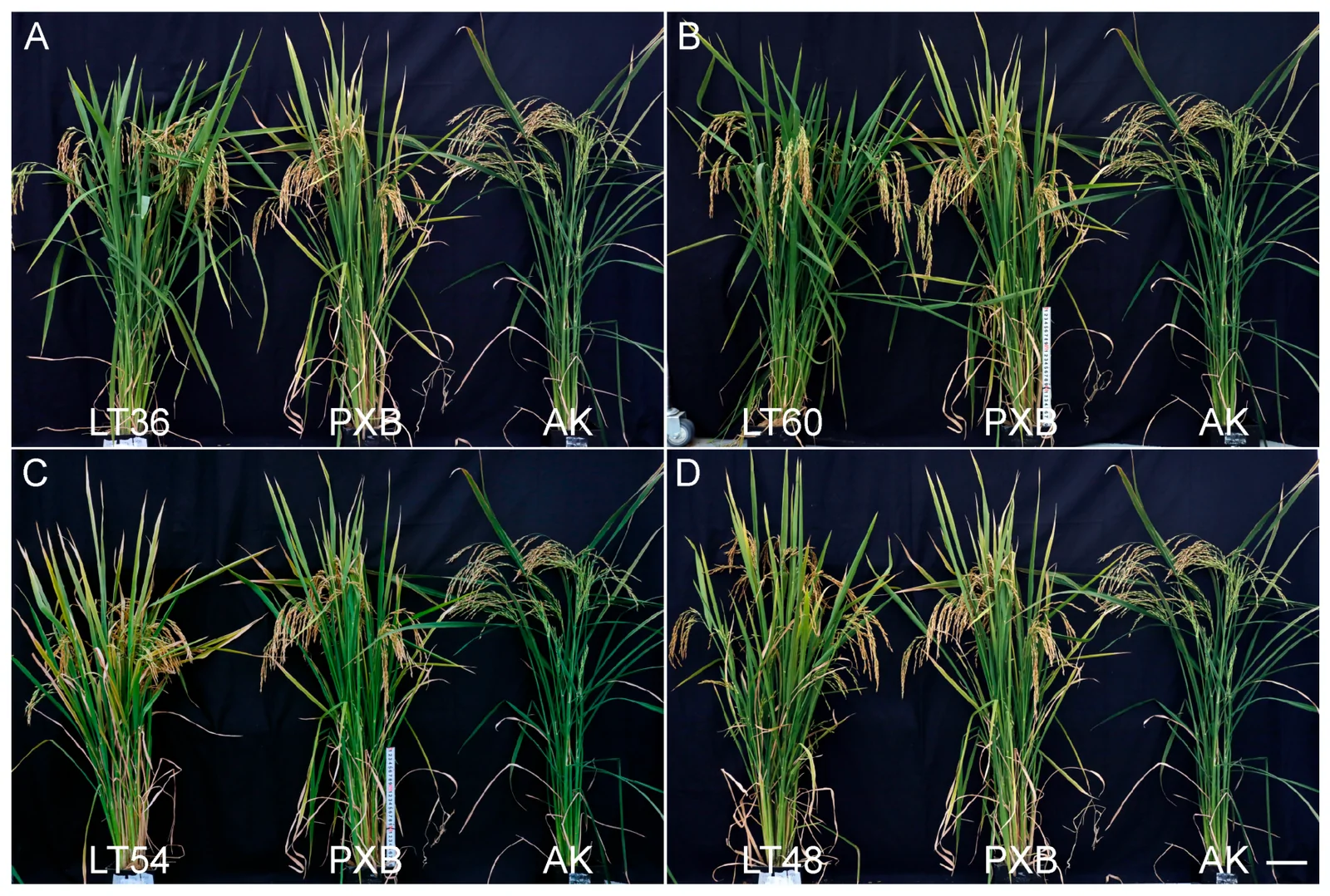
Phenotypes of backcross introgression lines at the VEQ2 locus. (**A**) Line LT36 (BC_3_F_4_); (**B**) Line LT60 (BC_2_F_5_); (**C**) Line LT54 (BC_2_F_2_); (**D**) Line LT48 (BC_3_F_3_). Lines LT36, LT60, and LT54 carry the CC genotype at VEQ2, whereas LT48 carries the TT genotype. PXB is the recurrent parent, and AKITAKOMACHI (AK) is the donor parent. Scale bar: 10 cm.

**Figure 4:**
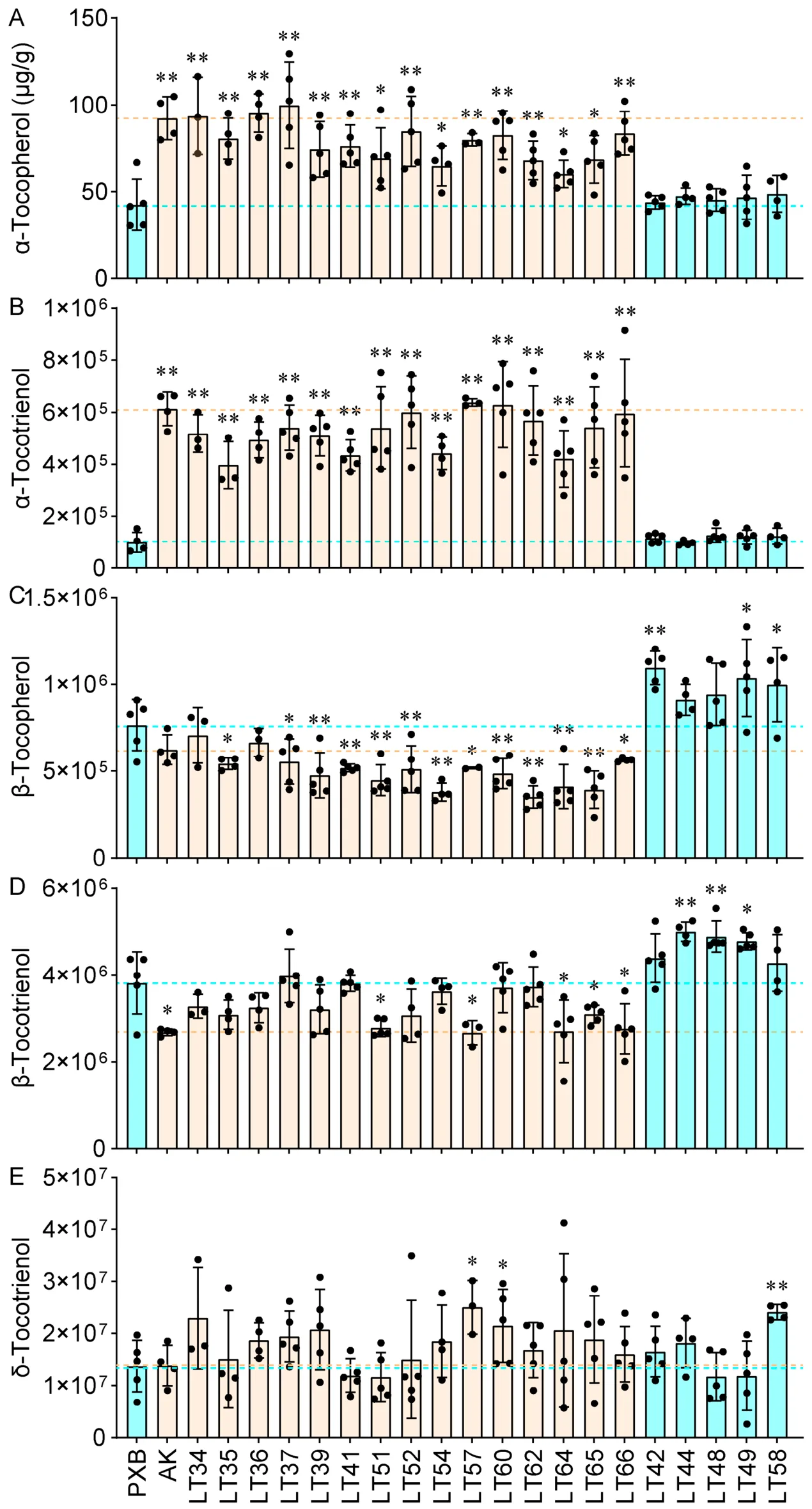
Composition analysis of vitamin E components in brown rice of backcross introgression lines at the VEQ2 locus. (**A**) Absolute content of α-tocopherol; (**B**) relative content of α-tocotrienol; (**C**) relative content of β-tocopherol; (**D**) relative content of β-tocotrienol; (**E**) relative content of δ-tocotrienol. PXB and AKITAKOMACHI (AK) are the recurrent and donor parent controls, respectively; LT34–LT66 are introgression lines. Blue and yellow dashed lines indicate the corresponding vitamin E component levels in PXB and AK, respectively; blue and yellow bars represent the TT and CC genotypes, respectively. Bar height represents the mean, error bars indicate standard deviation, and dots represent individual biological replicates. Asterisks indicate significant differences compared with the recurrent parent PXB: *, *p* < 0.05; **, *p* < 0.01 (Student’s *t*-test).

**Figure 5:**
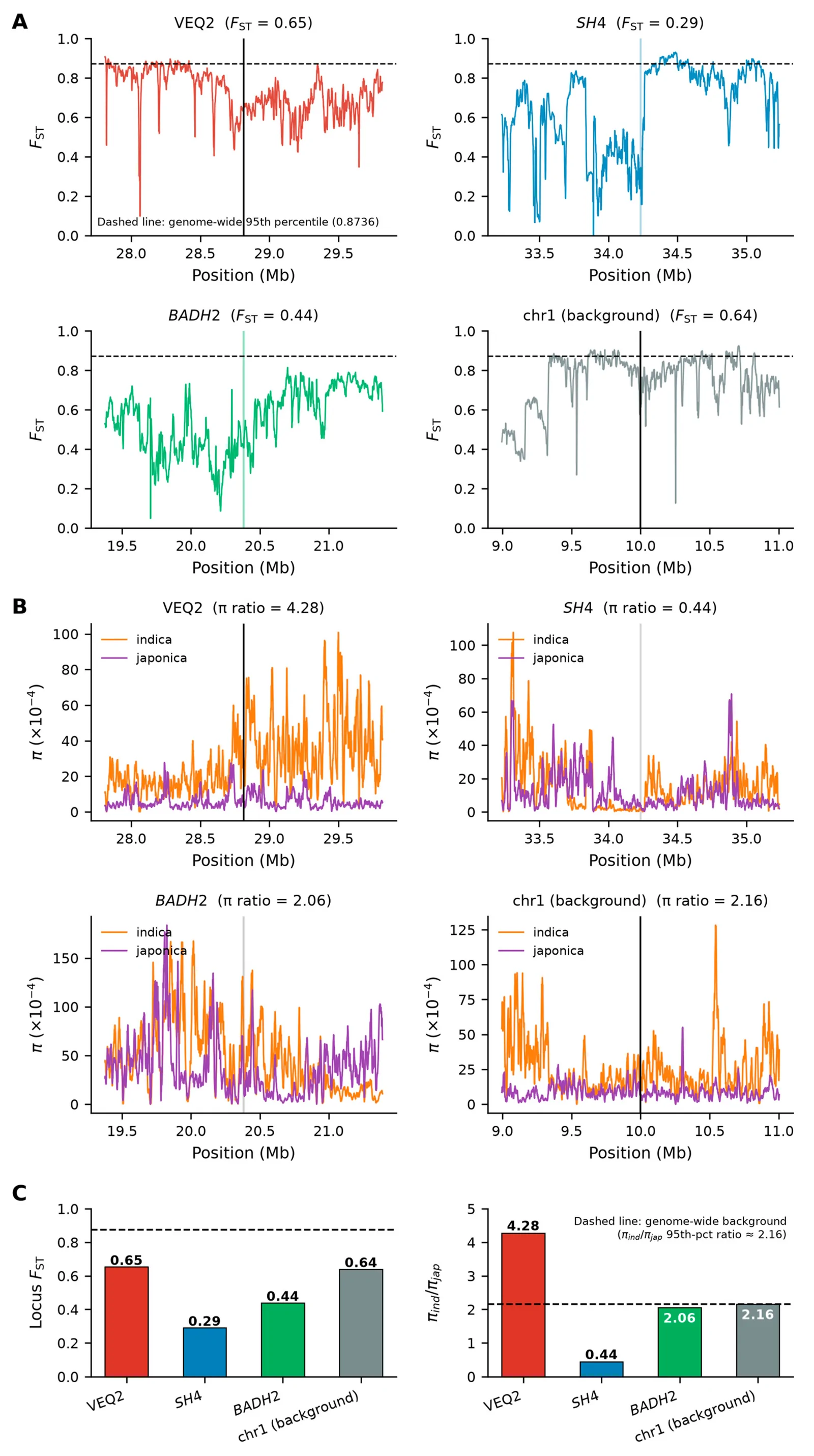
Population genetic statistics surrounding VEQ2 and comparative loci. (**A**) Weir–Cockerham weighted fixation index (*F_ST_*) between indica and japonica in 10 kb sliding windows (1 kb step) across the ±1-Mb regions surrounding VEQ2, *SH4*, *BADH2*, and a genomic background position (chr01:10,000,000). The dashed line indicates the genome-wide 95th percentile (0.8736); vertical lines mark the target SNP, gene, or background center of each locus. (**B**) Nucleotide diversity (π) of indica (orange) and japonica (purple) around VEQ2, *SH4*, *BADH2*, and the background position; vertical lines mark the target SNP, gene, or background center of each locus. (**C**) Locus-level *F_ST_* (**left**) and π_indica/π_japonica ratio (**right**) for the four loci; dashed lines indicate the genome-wide 95th percentile (*F_ST_*) and the background π ratio benchmark (~2.16). All statistics were calculated from RiceVarMap v3.0 genotype data of 4726 rice accessions.

**Table 1:** Agronomic traits of VEQ2 backcross introgression lines ^a,b,c,e^.

Line No. ^d^	PH (cm)	TN	SSR (%)	GL (mm)	GW (mm)	L/W	TGW (g)	YPP (g)
PXB	92.29 ± 1.8	12.43 ± 3.05	77.51 ± 2.90	7.52 ± 0.22	1.78 ± 0.07	4.3 ± 0.2	21.16 ± 0.09	26.19 ± 2.06
AK	114.57 ± 7.46 **	12.71 ± 1.98	78.31 ± 8.64	7.45 ± 0.31	1.71 ± 0.07 *	4.44 ± 0.1 *	20.9 ± 0.18 **	27.96 ± 5.08
CC	92.91 ± 5.23	14.77 ± 1.77	79.92% ± 2.57	6.37 ± 1.58	1.74 ± 0.19	3.70 ± 0.62	21.11 ± 0.10	25.85 ± 2.09
TT	95.43 ± 3.85	13.75 ± 0.72	79.12% ± 1.45	7.40 ± 0.18	1.72 ± 0.05	4.41 ± 0.08	21.15 ± 0.08	25.12 ± 3.43
LT34	103.29 ± 1.89 **	14.86 ± 2.54	87.65 ± 9.08 **	7.72 ± 0.51	1.87 ± 0.13	4.27 ± 0.18	20.95 ± 0.16 **	23.98 ± 3.4 *
LT35	96 ± 3.46 **	17.71 ± 3.73 **	79.75 ± 2.60	8.05 ± 0.5 *	1.88 ± 0.09 *	4.4 ± 0.14	21.21 ± 0.15	24.92 ± 2.99
LT36	92.57 ± 2.99	12.57 ± 2.37	81.13 ± 3.35 *	7.73 ± 0.68	1.85 ± 0.14	4.29 ± 0.17	21.19 ± 0.12	28.46 ± 3.61 *
LT37	98 ± 3.32 **	12.57 ± 1.62	79.62 ± 10.17	7.28 ± 0.41	1.71 ± 0.07 *	4.38 ± 0.32	21.18 ± 0.18	28.75 ± 2.59 **
LT39	96.14 ± 2.41 **	14.57 ± 2.37	80.84 ± 3.38 *	8.41 ± 0.46 **	2.02 ± 0.21 *	4.28 ± 0.34	21.18 ± 0.11	27.71 ± 4.22
LT41	99.43 ± 4.69 **	15.71 ± 1.89 *	80.14 ± 7.66	7.5 ± 0.5	1.8 ± 0.11	4.33 ± 0.26	21 ± 0.21 *	22.87 ± 4.77 *
LT51	90.29 ± 1.89 *	14 ± 3.21	77.47 ± 2.18	4.84 ± 1.37 **	1.6 ± 0.18 *	3.06 ± 0.69 **	21.12 ± 0.19	23.22 ± 5.01
LT52	91.29 ± 2.36	12.43 ± 1.81	79.05 ± 4.05	7.3 ± 1.13	1.93 ± 0.16 *	3.86 ± 0.35 *	21.23 ± 0.21	22.72 ± 2.66 **
LT54	87.14 ± 1.57 **	14 ± 1.29	75.93 ± 7.45	7.84 ± 0.77	1.98 ± 0.2 *	4.08 ± 0.44	21.21 ± 0.14	24.92 ± 3.41
LT57	90.43 ± 4.39	12.43 ± 0.98	80.41 ± 3.76 *	6.02 ± 1.43 **	1.83 ± 0.29	3.4 ± 0.5	21.2 ± 0.19	27.69 ± 8.52
LT60	89.43 ± 2.64 *	14.86 ± 1.35 *	80.07 ± 4.57	4.92 ± 0.79 **	1.54 ± 0.17 **	3.23 ± 0.3 **	21.04 ± 0.12 *	26.77 ± 3.52
LT62	86.86 ± 2.04 **	15.57 ± 1.4 *	77.38 ± 2.74	4.64 ± 0.92 **	1.52 ± 0.1 **	3.1 ± 0.5 **	21.12 ± 0.08	25.72 ± 5.88
LT64	87 ± 1.63 **	16.71 ± 1.98 **	79.48 ± 3.07	4.17 ± 0.53 **	1.48 ± 0.07 **	2.89 ± 0.27 **	20.97 ± 0.05 **	25.97 ± 3.61
LT65	NA	16.71 ± 1.5 **	80.56 ± 3.70 *	4.74 ± 0.88 **	1.59 ± 0.19 *	3.03 ± 0.32 **	21 ± 0.04 **	25.45 ± 3.08
LT66	NA	16.86 ± 2.27 **	79.26 ± 2.84	4.35 ± 1.42 **	1.52 ± 0.21 **	2.85 ± 0.58 **	21.01 ± 0.26	28.59 ± 4.4
LT42	98 ± 4.08 **	14 ± 1.53	79.30 ± 2.99	7.47 ± 0.43	1.78 ± 0.11	4.33 ± 0.31	21.22 ± 0.09	24.95 ± 7.03
LT44	NA	NA	78.45 ± 9.15	7.6 ± 0.4	1.74 ± 0.13	4.5 ± 0.24	21.04 ± 0.22	NA
LT48	97.29 ± 2.69 **	14.57 ± 1.99	78.59 ± 7.60	7.41 ± 0.28	1.71 ± 0.09	4.45 ± 0.23	21.11 ± 0.08	27.67 ± 6.43
LT49	96.71 ± 3.5 **	13.57 ± 1.72	77.75 ± 3.60	7.11 ± 0.58	1.64 ± 0.08 **	4.45 ± 0.21	21.19 ± 0.06	20.33 ± 2.93 **
LT58	89.71 ± 3.99	12.86 ± 1.35	81.53 ± 4.88 *	7.39 ± 0.58	1.75 ± 0.15	4.34 ± 0.21	21.2 ± 0.06	27.52 ± 8.14

^a^ Data are presented as mean ± standard deviation (SD) of 5–19 biological replicates. ^b^ PH, plant height; TN, tiller number; SSR, seed setting rate; GL, grain length; GW, grain width; L/W, length/width ratio; TGW, thousand grain weight; YPP, yield per plant. ^c^ Asterisks indicate significant differences compared with the control PXB (TT genotype) by Student’s *t*-test: * *p* < 0.05, ** *p* < 0.01. ^d^ PXB and AK are recurrent and donor parents, respectively. Values for CC and TT are means of CC- and TT-genotype introgression lines, respectively. LT34–LT66 are CC-genotype introgression lines; LT42–LT58 are TT-genotype introgression lines. ^e^ NA, sample not analyzed.

**Table 2:** Genotype frequency of the VEQ2 locus in 4726 rice germplasm accessions ^a,b^.

Populations ^c^	Size	Frequency of TT	Frequency of CC	Frequency of N
All	4726	52.7%	46.9%	0.4%
Indica	2759	84.2%	15.3%	0.6%
Japonica	1512	0.4%	99.5%	0.1%
Aus	269	49.8%	50.2%	0.0%
VI/Aromatic	96	1.0%	99.0%	0.0%
Intermediate	90	28.9%	67.8%	3.3%

^a^ Genotypes from RiceVarMap v3.0 (https://ricevarmap.ncpgr.cn/v3/ (accessed on 18 June 2026)). ^b^ CC and TT denote homozygous C and homozygous T genotypes, respectively. N indicates missing data at the VEQ2 locus. ^c^ Population classification follows RiceVarMap v3.0.

## Data Availability

All data and materials are available on request.

## Supplementary Materials

The following supporting information can be downloaded at: https://www.mdpi.com/article/10.3390/plants15192951/s1, Table S1: Summary of 533 rice accessions, including their population group, VEQ2 genotype, and grain α-tocopherol content; Table S2: Genotypes at the VEQ2 locus determined by Sanger sequencing in 13 rice varieties; Table S3: VEQ2 genotype and grain α-tocopherol content in F_2_ plants of Baikezaohe × AKITAKOMACHI; Table S4: Genotype at the VEQ2 locus, genetic background recovery rate, and grain vitamin E composition of backcross introgression lines; Table S5: Analysis of inter-generational variation in vitamin E component content among VEQ2 backcross introgression lines; Table S6: Population genetic statistics (*F_ST_* and π) at VEQ2 and comparative loci, with genome-wide 95th-percentile benchmarks; Table S7: Primers used in this study.

## Abbreviations

The following abbreviations are used in this manuscript:

GWAS	Genome-wide association study
KASP	Kompetitive allele-specific PCR
HGA	Homogentisate
PDP	Phytyl diphosphate
GGPP	Geranylgeranyl diphosphate
TAT	Tyrosine aminotransferase
HPPD	4-hydroxyphenylpyruvate dioxygenase
MEP	Methylerythritol phosphate
ABH	Alpha/beta hydrolase
PK	Phytol kinase
PKK	Phytyl phosphate kinase
HPT	Homogentisate phytyltransferase
MPBQ	2-methyl-6-phytyl-1,4-benzoquinol
HGGT	Homogentisate geranylgeranyltransferase
MGGBQ	2-methyl-6-geranylgeranylbenzoquinol
MPBQ-MT	MPBQ methyltransferase
TC	Tocopherol cyclase
γ-TMT	γ-tocopherol methyltransferase
RDA	Recommended dietary allowance
CV	Coefficient of variation
SNP	Single nucleotide polymorphism
a	Additive
d	Dominance
*F_ST_*	Fixation index
MAF	Minor allele frequency
EMMAX	Efficient mixed-model association expedited
